# Fault Diagnosis Method of Special Vehicle Bearing Based on Multi-Scale Feature Fusion and Transfer Adversarial Learning

**DOI:** 10.3390/s24165181

**Published:** 2024-08-10

**Authors:** Zhiguo Xiao, Dongni Li, Chunguang Yang, Wei Chen

**Affiliations:** 1 School of Computer Science & Technology, Beijing Institute of Technology, Beijing 100811, China; ldn@bit.edu.cn; 2 College of Computer Science and Technology, Changchun University, Changchun 130022, China; 3 National Key Laboratory of Special Vehicle Design and Manufacturing Integration Technology, Baotou City 014030, China; chunguang33@126.com (C.Y.); 18686131380@163.com (W.C.)

**Keywords:** rolling bearing, fault diagnosis, multi-scale feature extraction, transfer learning

## Abstract

To address the issues of inadequate feature extraction for rolling bearings, inaccurate fault diagnosis, and overfitting in complex operating conditions, this paper proposes a rolling bearing diagnosis method based on multi-scale feature fusion and transfer adversarial learning. Firstly, a multi-scale convolutional fusion layer is designed to effectively extract fault features from the original vibration signals at multiple time scales. Through a feature encoding fusion module based on the multi-head attention mechanism, feature fusion extraction is performed, which can model long-distance contextual information and significantly improve diagnostic accuracy and anti-noise capability. Secondly, based on the domain adaptation (DA) cross-domain feature adversarial learning strategy of transfer learning methods, the extraction of optimal domain-invariant features is achieved by reducing the gap in data distribution between the target domain and the source domain, addressing the call for research on fault diagnosis across operating conditions, equipment, and virtual–real migrations. Finally, experiments were conducted to verify and optimize the effectiveness of the feature extraction and fusion network. A public bearing dataset was used as the source domain data, and special vehicle bearing data were selected as the target domain data for comparative experiments on the effect of network transfer learning. The experimental results demonstrate that the proposed method exhibits an exceptional performance in cross-domain and variable load environments. In multiple bearing cross-domain transfer learning tasks, the method achieves an average migration fault diagnosis accuracy rate of up to 98.65%. When compared with existing methods, the proposed method significantly enhances the ability of data feature extraction, thereby achieving a more robust diagnostic performance.

## 1. Introduction

Special vehicles, including military and engineering vehicles, frequently operate in extreme conditions such as heavy loads, high speeds, temperature extremes, dust, and mud. Rolling bearings are among the most vulnerable components in rotating machinery. Therefore, early fault diagnosis of rolling bearings is crucial. Traditional solutions for diagnosing rolling bearing faults involve using sensors to collect bearing fault signals, extracting relevant fault characteristics, and classifying the faults [1]. Previous studies in the field of special vehicle bearing fault diagnosis have achieved certain results [2]. Traditional bearing fault diagnosis methods primarily rely on vibration signal analysis [3]. By collecting vibration signals during bearing operation and extracting time domain, frequency domain, and time–frequency domain characteristics, the accuracy of fault classification can be directly influenced. Signal processing methods, such as the Hilbert transform [4], Fourier transform, empirical mode decomposition [5], and wavelet transform [6], can extract fault characteristics from both linear and nonlinear signals. Deep learning models are also applicable to bearing data as they can directly process various types of raw data [7]. To develop a fully interpretable network, Li et al. [8] propose a transparent operator network incorporating a parameterized signal operator node. This node uses a learnable Morlet wavelet operator in the frequency domain, with signal-wise gated matrices and skip connections. By stacking multi-channel and multilayered nodes as signal operator layers and integrating statistical features with a linear classifier, the resulting modules are physically interpretable. Qin et al. [9] introduced a dynamic weighted federated Remaining Useful Life (RUL) prediction framework, consisting of a cloud server and multiple edge clients. This framework aggregates into a global model through edge–cloud collaboration and dynamic weighting. Experimental results on gears and datasets validate the proposed method’s effectiveness and superiority. These studies offer pioneering approaches to enhancing diagnostic models’ interpretability and fault prediction through edge–cloud collaboration, serving as valuable references for future research.

With the continuous development of machine learning, certain algorithms can explore deeper levels of potential information and utilize it for self-feedback, thereby achieving desirable predictive analysis results [10]. For instance, applying fault features to machine learning algorithms such as decision trees [11], Bayesian classifiers [12], support vector machines [13], and artificial neural networks (ANNs) [14] can effectively classify faults. Deep learning is a machine learning method based on neural networks that automatically extracts features and performs classification or regression by learning from large amounts of data. In recent years, deep-learning-based fault diagnosis methods have become a major approach in fault diagnosis and health maintenance technology [3]. In rolling bearing fault diagnosis, deep learning can train models to learn the characteristics of vibration signals and classify faults based on these characteristics. Current research generally focuses on signal processing and feature extraction for rolling bearings, as well as the construction of fault diagnosis models and the study of model robustness.

Although significant progress has been made in the diagnosis technology of special vehicle bearing faults, there are still some challenges and problems. Due to the lack of rolling bearing fault samples in the actual working environment, as well as the influence of environmental noise and load changes, the distribution of fault samples varies, resulting in poor generalization of diagnosis. Lv Huan et al. [15] proposed a rolling bearing fault diagnosis method based on improved DenseNet and transfer learning. The cross-domain diagnosis accuracy under small sample and variable load conditions has reached over 90%, which has better generalization compared to other models. Different types of faults in special vehicles may produce similar signal characteristics, while the same type of fault may also exhibit different characteristics under different working conditions. To achieve cross-condition and cross-domain fault diagnosis, some scholars have proposed deep transfer models that can adaptively align data from different domains, facilitating transfer learning and yielding good results [16]. Data-driven bearing fault diagnosis methods have shown superior performance, but it is difficult to collect a large amount of data in the actual production process, and the value of bearing fault diagnosis based on small samples is highlighted [17].

To address the aforementioned challenges and issues, this study aimed to explore more accurate and efficient techniques for diagnosing bearing faults in special vehicles and offer theoretical and technical support for their application in real-world environments. Specifically, this paper proposes a rolling bearing diagnostic method based on multi-scale convolutional feature fusion and transfer adversarial learning. The specific contributions include the following:

⚫ A multi-scale feature fusion network with an attention mechanism is designed. This network implements one-dimensional multi-scale convolution and position encoding of the original bearing data through multi-scale one-dimensional convolutional network modules, capturing the remote dependence information of variable-length sequence data, thereby achieving deeper multi-scale feature fusion and extraction;
⚫ A cross-domain feature adversarial transfer learning method for domain adaptation (DA) is developed. This method achieves optimal domain-invariant feature extraction by reducing the gap between the target domain and the source domain distributions, addressing the issues of cross-condition, cross-device, and cross-virtual–real transfer in fault diagnosis of special vehicle bearings;
⚫ Comparative experiments demonstrate that the proposed method significantly outperforms existing mainstream methods in terms of its feature extraction capability and data transfer diagnosis.

## 2. Related Work

Various types of bearing faults, including rolling element faults, inner ring faults, and outer ring faults, exhibit distinct characteristics at different scales. Traditional machine learning methods often require complex feature engineering when processing bearing data, while deep learning methods can automatically extract features, but are limited by the quantity and quality of labeled data. Multi-scale feature fusion can better adapt to different fault modes and effectively recognize various fault types. In addition, in some specific bearing fault diagnosis tasks, there may be a problem of insufficient available data, which can lead to overfitting and poor generalization ability of the model. A better solution is to use transfer learning methods to address the issue of different bearings operating in varied environments, facilitating cross-domain knowledge transfer. This allows the application of knowledge learned in one field to another, thereby enhancing the model’s applicability.

### 2.1. Multi-Scale Feature Extraction and Fusion Method for Bearing Raw Data

The vibration signals generated by bearings during operation are often very complex, containing multiple frequency components and noise. The processing of bearing vibration data is a comprehensive technical process aimed at extracting useful information from the complex vibration signals generated during the operation of bearings. Through multi-scale feature extraction, the essential characteristics of the signals can be captured from different perspectives and at different scales, including instantaneous characteristics, periodic changes, the spectral distribution, and so on.

The raw data of rolling bearings for special vehicles collected by sensors usually contain a large amount of noise and interference, so preprocessing is required, including filtering, denoising, normalization, etc., to improve data quality [5]. Basic statistical features of the data, such as mean, variance, peak, etc., are extracted through time domain analysis, which can reflect the fluctuation and pulse conditions of the vibration signal. Since the bearing vibration signal may be non-stationary and nonlinear, further time–frequency domain analysis such as wavelet transform and short-term Fourier transform can provide frequency information that changes over time, which is crucial for capturing transient or rapidly changing fault characteristics [4]. That involves a process of using empirical mode decomposition to decompose the signal into the intrinsic mode function (IMF), then performing bispectrum analysis on the IMF signal, and finally, extracting the bearing fault characteristic frequency to achieve fault diagnosis. This method may suffer from mode mixing, be sensitive to noise, and the decomposition results may lack consistency [18]. Optimizing variational modal decomposition through genetic algorithms, and then determining the type of bearing fault through the envelope spectrum of the modal components, requires setting the number of modes and regularization parameters, and parameter selection has a significant impact on the results [19].

As a nonlinear method, multi-scale permutation entropy is widely used in the evaluation of the complexity and randomness of time series. Li Yongjian et al. [20] proposed an improved multi-scale permutation entropy method for bearing data. By comparing the simulated signal with the traditional multi-scale permutation entropy method, it was found that the entropy estimation results of the improved multi-scale permutation entropy method were more stable and the error was reduced at different scales. However, in practical use, the method is sensitive to parameter settings and noise. In addition, with the development of machine learning and deep learning technology, some researchers have also tried to use these methods to automatically extract multi-scale features of bearing data, and proposed a bearing fault diagnosis method based on parallel convolutional neural network (CNN) multi-scale feature fusion [21].

Another study uses bearing vibration data from different perspectives as inputs to parallel CNNs, and trains classification modules through high-level fault features to achieve bearing fault diagnosis. The Markov transition field (MTF) encoding method is used to convert the original one-dimensional vibration signal into a two-dimensional feature image with temporal correlation, and then the feature map is used as the input of the convolutional neural network (CNN) for automatic feature extraction and fault diagnosis [22]. In addition, some new concepts such as deep separable convolutional neural networks have also been proposed. This divides the convolution process into depthwise convolution and pointwise convolution on the basis of the original neural network. The separable neural network can achieve the same effect as ordinary CNNs, with fewer total parameters and a higher learning efficiency, thus achieving a lightweight network structure while ensuring accuracy [23].

The extraction of multi-scale features from time-series data can reduce computational costs, integrate information from different scales, and provide richer feature representations, thereby improving the performance of classification, recognition, and other tasks. Existing CNNs use single-scale convolution kernels to extract features, but they cannot capture the multi-scale features of vibration signals. Among existing models that can extract multi-scale features, most simply stack the obtained features and perform fault diagnosis without considering the different weight effects of multi-scale features in fault diagnosis. The encoding and representation learning of input sequences cannot solve the long-term dependence problem between the input and output. The model lacks consideration of parallel computing capabilities and consumes a lot of computing resources.

### 2.2. Bearing Fault Diagnosis Based on the Transfer Learning Method

Due to the small number of fault samples with labels in the bearing fault diagnosis, and the domain shift problem between the source domain data and the target domain data, the accuracy of bearing fault diagnosis is greatly reduced. Zhu Xudong [24] proposed an iterative diagnosis method for bearing faults based on improved balanced distribution adaptation transfer learning. Starting from the analysis of the fault signal characteristics of rolling bearings’ structures and parts, an improved balanced distribution adaptation method was proposed to solve the problem of heterogeneous domain adaptation caused by unknown differences in edge distribution and conditional distribution. The target domain pseudo-label iterative optimization method based on transfer learning and the KNN algorithm was designed to determine the fault labels of target domain samples, achieving heterogeneous sample fault diagnosis. Hu Ruohui et al. [25] proposed an effective transfer learning model that utilizes a small amount of sample data to achieve domain adaptation. The simulated expansion of a small numberof vibration signals was achieved through deep convolutional generative adversarial networks (DCGANs), and the source and target domain features were projected into the same feature space through domain-adversarial neural networks (DANNs), achieving multi-domain feature extraction and adaptation, thereby enabling the diagnosis network to identify the health status of unknown-labeled rolling bearings under varying operating conditions. Ma et al. [26] proposed a lightweight bearing fault diagnosis method based on depthwise separable convolutional neural networks (DSCNNs). This method has good transfer learning and data-driven capabilities, and it can achieve good diagnostic results faster and more lightly during the diagnosis process. It has been verified that by adding a DSCNN to the feature learning model, the learning ability and generalization ability of the bearing fault diagnosis model can be improved, effectively improving the accuracy of bearing fault diagnosis [27].

Due to the substantial differences in the working environments of rolling bearings in special vehicles, effective feature extraction is crucial for bearing fault diagnosis. To enhance the ability of transfer learning models to extract effective high-level features from raw data and adapt across different domains, it is necessary to address the challenges of cross-condition transfer, cross-device transfer, and cross-virtual–real transfer in bearing diagnosis for various types of special vehicles.

## 3. Materials and Methods

**Figure 1:**
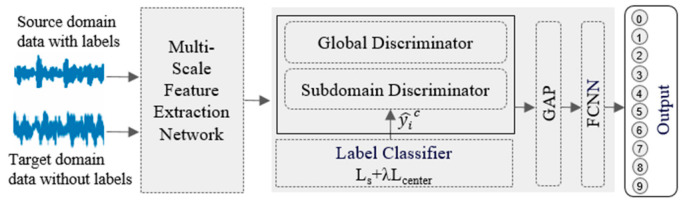
Domain adversarial network model with multi-scale feature fusion.

In order to solve the problem of difficult-to-achieve effective fault diagnosis of unlabeled data under different working conditions in the process of transfer learning, we propose a domain-adversarial network for multi-scale feature fusion (DNMFF), for intelligent fault diagnosis of rolling bearings in special vehicles. The established DNMFF’s model diagram is shown in Figure 1.

**Figure 2:**
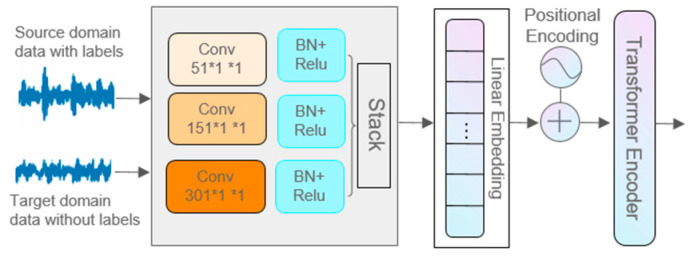
Multi-scale feature extraction network.

The network mainly includes two parts: multi-scale feature extraction and fusion, and an adaptive adversarial network. In the multi-scale feature extraction and fusion part, a 1D-CNN with multi-scale convolution kernels is used to automatically extract feature sets from the signal in the source domain and target domain, perform encoding operations, and input them into an improved Transformer Encoder for parallel multi-head, multi-scale feature extraction and feature fusion, as shown in Figure 2. In the adaptive domain adversarial network part, a dynamic domain adversarial learning strategy based on transfer learning is adopted. The proportions of edge distribution and conditional distribution in the transfer learning process are reasonably adjusted according to the similarity between working conditions, reducing the distribution difference between the source domain and target domain, thus achieving the purpose of transferring the “prior knowledge” of the source domain to the target domain. In order to effectively train model parameters, a gradient reversal layer is added to the conditional distribution discriminator and edge distribution discriminator. GRL [28] achieves automatic reversal of the gradient direction in backpropagation, while maintaining the gradient direction unchanged in forward propagation. During the application of the model, the rolling bearing data of special vehicles are used as inputs, which enter the scale feature extraction and fusion module. After being assessed by the subdomain discriminator, they are connected to the global max-pooling layer (GAP) and fully connected layer (FCNN), and the classification result is obtained through the classifier.

### 3.1. Multi-Scale Feature Extraction and Fusion Network

As shown in Figure 2, the structure of the multi-scale feature extraction and fusion network includes five layers, which are the input layer, one-dimensional convolutional layer, linear transformation layer, position encoding layer, and the multi-scale feature fusion extraction layer based on the Transformer encoding structure.

(1) Data Input

Both source domain data and target domain data play a role in the training phase to enhance the model’s performance in the target domain. Source domain data typically have rich annotation information and are used to train an initial model. The raw vibration signals of rolling bearings across domains serve as both source domain data and target domain data, which can be directly input into the network for training. In the model usage phase, only target domain data are input into the model for feature extraction and inference.

(2) One-dimensional Convolutional Layer

A convolutional neural network (CNN) [7] is a type of neural network specifically designed for processing data with a known, grid-like topology. It has achieved great success in practical applications, especially in the field of image classification [15]. The network structure consists of layered, trainable layers, mainly including the input layer, convolutional layer, activation layer, pooling layer, and fully connected layer, which can learn effective feature representations [16]. A typical convolutional layer for image classification involves input digital image *I* and convolutional kernel *K*, and the mathematical model of convolution can be defined as follows:

(1) $$S(i,j)=(I*K)(i,j)=\sum_{m}\sum_{n}I\left(m,n\right)K\left(i-m,j-n\right),$$

The data to be processed in this paper are one-dimensional time domain vibration signals, which require the application of one-dimensional convolution in each convolutional layer for data processing. When *m* in Formula (1) is 1, one-dimensional convolution is obtained, which can be represented as follows:

(2) $$C_{ij}^{l}=\emptyset \left(k_{n\times 1}^{j}*x_{i:i+n}^{i}+b_{ij}\right),$$

Rich feature representation extraction is achieved through convolution operations with multiple one-dimensional convolutional kernels. One-dimensional convolution performs convolution on the input data. Specifically, the input signal is denoted as $X\in R^{W\times 1\times 1}$, where *W* is the length of each input. The multi-scale convolution fusion layer contains multiple convolutions with kernels of different sizes. The sizes of the convolutional kernels are hyperparameters set by the network, and three different scales can be used according to different inputs. Then, through multi-channel pooling operations and stacking of each feature component, multi-scale features are obtained.

(3) Linear Transformation Layer

Before inputting the multi-scale features into the linear embedding layer, this paper extracts data with a length of P in each dimension, forming a total of P×1×3 layers, and creates a series of signal patches $x=\left(x_{1},x_{2},\ldots ,x_{m}\right)$ with a length of *m*. When m=W/P, these patches are then linearly projected into vectors with a model dimension of D using the learned embedding matrix $E\in R^{3PC\times D}$. The embedded representations are concatenated with learnable class tokens x_class for further feature extraction. Through linear transformation, the neural network can extract useful features from the input data and represent them in a more manageable form.

(4) Position Encoding Layer

The self-attention layer needs to capture positional information in the signal sequence. Therefore, to maintain the same spatial arrangement as the original vibration signal after data encoding, positional information $E_{pos}\in R^{\left(m+1\right)\times D}$ with dimension d is encoded and appended to the signal patches x. The generated embedding sequence with token $z_{0}$ is represented as follows:

(3) $$z_{0}=\left[x_{class};x_{1}E;\ldots ;x_{m}E\right]+E_{pos},$$

(5) Feature Fusion Module Based on a Modified Transformer

**Figure 3:**
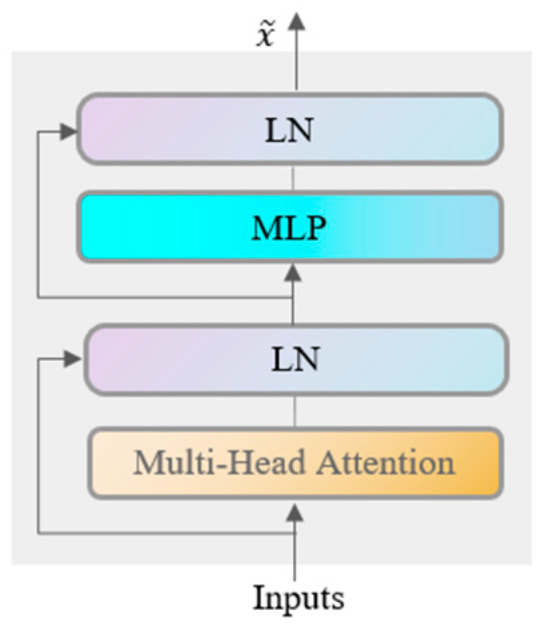
Feature encoding fusion module structure diagram.

The Transformer originates from a classic article published by Google in 2017 titled “Attention is all you need” [29], which is fundamentally structured as an Encoder–Decoder architecture. The encoding component is composed of multiple layers of Encoders, and the decoding component is composed of the same number of layers of Decoders. The Transformer Encoder part is the Encoder module of the Transformer. The method in this paper introduces the Encoder module of the Transformer to process contextual information in the input sequence and perform real-time feature extraction on the one-dimensional input sequence of rolling bearings. Since existing research and applications do not involve mapping the new representation space generated by the Encoder back to the sequence output, this paper directly removes the entire Decoder part. Based on the retained Encoder part, it is designed as a multi-scale feature encoding fusion module for the existing model. This module can capture global information in the input data sequence of rolling bearings and obtain hidden representations of each input scale datum. Its structure is shown in Figure 3.

The feature encoding fusion module is composed of modules such as the multi-head attention mechanism, residual connection with layer normalization (LN), and multilayer perceptron (MLP). By stacking N identical Encoders, relevant feature information from the data is extracted. This enhances the flow of information to achieve higher performance. The output of these operations can be represented as $\tilde{x}$, as shown in Formula (4):

(4) $$\tilde{x}=x+Multihead(LN(x)),$$

Here, *x* is the input to the Encoder, that is, the data encoded by the positional encoding layer as time-series data. Multi-head attention is equivalent to having multiple sets of attention mechanisms [29]. The processing method for each head in the multi-head attention mechanism is the same as that in the self-attention mechanism. Specifically, the input vector X is multiplied by the weight matrices $W^{Q}$,$\;W^{K}$, and $W^{V}$, respectively, to obtain the *Q* (Query) matrix representing the input vector at the current time, and the *K* (Key) matrix representing the information at all times in the input sequence, which is usually obtained through a linear transformation of the input vector and the key weight matrix. The *V* (Value) matrix also represents the information at all times in the input sequence; its main function is to generate the final output through a weighted sum with the query results, as expressed in Equation (5):

(5) $$Attention\left(Q,K,V\right)=Softmax\left(\frac{QK^{T}}{\sqrt{d_{k}}}V,\right)$$

In the formula, $\frac{QK^{T}}{\sqrt{d_{k}}}$ represents the dot product of the Query and Key matrices divided by the square root of the dimension of the Key vectors. The Softmax function is used to normalize and obtain the attention weights. Finally, the results are concatenated to achieve the mapping of multiple sets of parameter matrices to different vector spaces and to integrate the results from these different vector spaces.

### 3.2. Domain Adversarial Network Diagnostic Model Based on Transfer Learning

To address the challenges of transfer learning in rolling bearing fault diagnosis for special vehicles under different operating environments, we have designed a deep transfer diagnostic model based on a domain adversarial network. In this model, we use the bearing fault dataset from Case Western Reserve University as the source domain and data from a simulated special vehicle bearing test bench as the target domain to jointly train and optimize various parameters in the domain adversarial network diagnostic model. As can be seen in Figure 1, the model structure mainly consists of three parts: a domain discriminator composed of a source domain discriminator and a target domain discriminator, and a fault label classifier aimed at distinguishing bearing fault states.

#### 3.2.1. Label Classifier Using a Hybrid Loss Function

**Figure 4:**
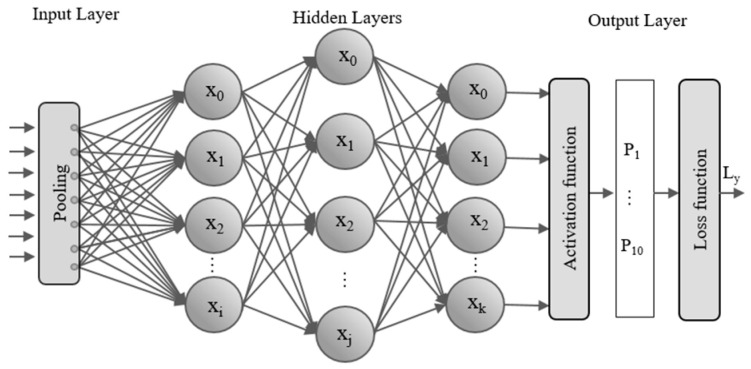
Label classifier network architecture diagram.

In the diagnostic model, the label classifier is composed of fully connected layers and a loss function, realizing the function of classifying input data that can reflect bearing fault characteristics. The data are input into the fully connected layers, which can reduce the distributed feature representations learned by the multi-scale feature fusion extraction module from high dimensions to low dimensions and map them into the existing fault label space. This process obtains the score of the input data in each bearing fault category. Through the calculation of the activation function and loss function, the probability output and corresponding loss of each fault category can be obtained. The backpropagation method is used to reduce the loss by training the parameters in the network, aiming to reduce prediction errors and improve classification accuracy. The specific structure of the label classifier is shown in Figure 4.

For common multi-class classification problems, the loss is often calculated using the Softmax loss function. The definition of the Softmax function is given in Formula (6):

(6) $$S_{i}\left(x_{i}\right)=\frac{x_{i}}{\sum_{j=1}^{n}x_{j}}\;\;\;\;\;\;\;i=1,2,\ldots \ldots ,n,$$

where $x_{i}=W_{i}^{T}x+b_{i}$ is the ith output of the full connection layer, that is, the prediction result of the input data in the *i*-th category. The definition of the Softmax loss function is given in Formula (7):

(7) $$L_{s}=-\sum_{i=1}^{N}\left(P_{i}{log}_{2}(s(x_{i}))\right),$$

In the formula, N represents the number of samples in the input batch, i represents the number of categories, and $P_{i}$ is a 1×T vector containing T values, where the value at the position corresponding to the true label is 1, and the others are 0.

However, when using the Softmax loss function to calculate the loss, only the distance between classes is considered. Although clear boundaries between classes can be observed overall, there may be cases of misclassification for individual samples when the feature centers of two classes are close to each other. To address this issue, a center loss function is introduced based on the Softmax loss function to form a hybrid loss function. The definition of the center loss function is given in Formula (8):

(8) $$L_{center}=\frac{1}{2}\sum_{i=1}^{m}∥x_{i}-c_{y_{i}}{∥}_{2}^{2},$$

where $x_{i}$ represents the feature vector of the *i*-th sample, $c_{y_{i}}$ represents the feature center of the class $y_{i}$ that the *i*-th sample belongs to, and m is the batch size of the training data. The center loss function calculates the sum of squared Euclidean distances between the sample feature vectors and the feature centers of their respective classes for a batch of data. A smaller value of $L_{center}$ indicates that the sum of squared distances between each batch of samples and the class feature centers is smaller, meaning that the intra-class data are more compact and the distances between individual data within each class are reduced. The Softmax loss and the center loss can be combined with a certain weight relationship to form a hybrid loss function, as shown in Formula (9):

(9) $$L_{y}=L_{s}+\gamma L_{center}=-\sum_{i=1}^{N}\left(P_{i}{log}_{2}(s(x_{i}))\right)+\frac{\gamma }{2}\sum_{i=1}^{m}∥x_{i}-c_{y_{i}}{∥}_{2}^{2},$$

Utilizing a hybrid loss function instead of the Softmax function in the label classifier can enhance intra-class compactness while maintaining clear inter-class boundaries and increased distances. This improvement enhances the label classifier’s ability to distinguish extracted features, thereby increasing the robustness of the diagnostic model.

#### 3.2.2. Domain Discriminator for Adapting Marginal and Conditional Distributions

The domain adversarial neural network (DANN) is a special network architecture in the field of deep transfer learning, inspired by generative adversarial networks (GANs) [30]. It is designed to optimize the training process of diagnostic models, enabling them to learn transferable features more effectively. This network architecture consists of three key components: a feature extractor G, a domain discriminator D, and a label classifier Y. The primary responsibility of the feature extractor G is to extract shared features between the source domain and the target domain, while the domain discriminator D strives to distinguish whether these features originate from the source domain or the target domain. To achieve a balance between these two components in the adversarial process, a special structure called the gradient reversal layer (GRL) is introduced between the feature extractor and the domain discriminator. This layer performs an identity transformation during forward propagation but automatically reverses the gradient direction of the domain classification loss during backpropagation, thereby influencing the update of the feature extractor’s parameters.

Unlike the traditional DANN model [31], the domain adversarial network proposed in this paper aligns both marginal and conditional distributions during domain adaptation. Given that the contributions of these two distributions vary across different tasks, this method employs an adaptive weight factor to dynamically allocate their proportions. This approach significantly enhances the model’s ability to adapt to new tasks or data.

The domain discriminator constructed in this paper consists of two parts: a global domain discriminator and a subdomain discriminator. These two components are responsible for adapting the marginal distribution and the conditional distribution between the source domain and the target domain, respectively. In the framework of domain adaptation, global and subdomain discriminators are two commonly used types of discriminators. There is an inclusion relationship between them: the global domain discriminator serves as the macroscopic entity of the subdomain discriminator, while the subdomain discriminator can be regarded as a specific refinement of the global domain discriminator. When performing domain adaptation, we typically need to handle scenarios where the source domain and the target domain have different data distributions. Formula (10) describes the loss incurred when the shared features of the source and target domains are input into the global domain discriminator.

(10) $$L_{g}=\frac{1}{n_{t}+n_{s}}\sum_{x_{i}\in D_{s}\cup D_{t}}L_{s}(\hat{D}\left(G\left(x_{i}\right)\right),d_{i}),$$

where G(·) represents the feature extraction process, $\hat{D}\;(\cdot )$ represents the global domain discrimination process, $L_{s}\;(\cdot )$ represents the Softmax loss function, $d_{i}$ represents the domain label (0 for the source domain, 1 for the target domain), $n_{s}$ represents the number of samples in the source domain, and $n_{t}$ represents the number of samples in the target domain. Before the shared features of the source and target domains are input into their corresponding subdomain discriminators, they need to be multiplied by the category probabilities ${\hat{y_{i}}}^{c}$ obtained from the label classifier to determine the weight of each subdomain discrimination. The calculation of the loss is shown in Formula (11):

(11) $$L_{l}=\frac{1}{n_{t}+n_{s}}\sum_{c=1}^{C}\sum_{x_{i}\in D_{s}\cup D_{t}}L_{s}^{c}(\overset{‿}{D^{c}}\left({\hat{y_{i}}}^{c}G\left(x_{i}\right)\right),d_{i}),$$

In Formula (10), $\overset{‿}{D^{c}}$ represents the discriminator for the *c*-th subdomain, and $L_{s}^{c}$ represents the Softmax loss function for the *c*-th subdomain. From this, the loss of the entire domain discriminator can be obtained, as shown in Formula (12):

(12) $$L_{D}=L_{g}+\sigma L_{l},$$

The weight factor σ can be solved based on the distribution distance between the source domain and the target domain, using A−Distance as the measurement criterion. The marginal distribution distance between the source domain and the target domain obtained by the global domain discriminator is given by Formula (13):

(13) $$d_{A,g}\left(D_{s},D_{t}\right)=2(1-2(L_{g})),$$

The conditional distribution distance between the source domain and the target domain obtained by the subdomain discriminator is represented as follows:

(14) $$d_{A,l}\left(D_{s}^{c},D_{t}^{c}\right)=2(1-2(L_{l}^{c})),$$

The weight factor σ can be represented by Formula (15):

(15) $$\sigma =\frac{d_{A,g}\left(D_{s},D_{t}\right)}{d_{A,g}\left(D_{s},D_{t}\right)+\frac{1}{C}\sum_{c-1}^{C}d_{A,l}\left(D_{s}^{c},D_{t}^{c}\right)},$$

The goal of the global domain discriminator is to discriminate between the source domain and the target domain, in order to distinguish the feature differences between the two domains. The global domain discriminator can be trained as a binary classifier to determine whether a sample comes from the source domain or the target domain. The subdomain discriminator, as part of the global domain discriminator, aims to discriminate within the target domain to distinguish different categories within it. The subdomain discriminator can be trained as a multi-class classifier to determine which category a target domain sample belongs to. Through iterative adversarial training, the model parameters gradually converge, allowing the model to extract common features between the source and target domains by confusing their data, and to learn a relatively optimal weight factor that reflects the ratio of marginal distribution and conditional distribution between the source and target domains. Ultimately, the loss of the entire diagnostic model can be obtained, as shown in Formula (16),

(16) $$L\left(\theta_{f},\theta_{y},\theta_{d},\theta_{d}^{c}{|}_{c=1}^{C}\right)=L_{y}-\alpha L_{D}$$

where $\theta_{f}$ represents the parameters of the feature extractor, $\theta_{y}$ represents the parameters of the label classifier, $\theta_{d}$ represents the parameters of the global domain discriminator, $\theta_{d}^{c}{|}_{c=1}^{C}$ represents the parameters of the subdomain discriminators for each category (with *C* being the total number of categories), and α represents the weight of the domain discrimination loss.

## 4. Experimental Analysis

### 4.1. Experimental Environment

**Figure 5:**
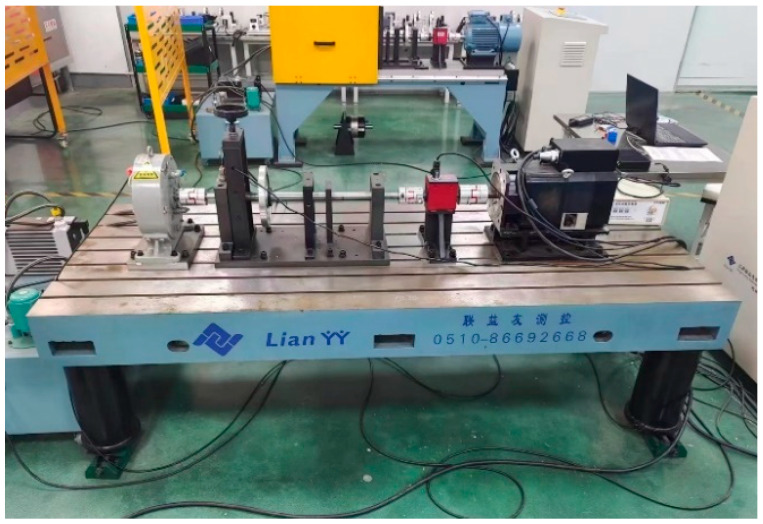
Special vehicle rolling bearing fault simulation test bench.

The data in the experiment were divided into two categories. The source domain data were those of the bearing dataset from Case Western Reserve University, which included vibration signals of rolling bearings in four states: normal condition, outer race fault, roller fault, and inner race fault. The target domain data were collected from a special vehicle bearing test bench in a simulated laboratory environment, as shown in Figure 5.

The two datasets had the same types of faults. During the experiment, we extended the training samples by slicing overlapping raw signals to achieve data augmentation of the original data. The specific method was to set the length of each sample to 1024 and the shift size between two adjacent samples to 512. The dataset contained 3600 training samples and 400 test samples, with four different health states.

In the experiment, the implementation language was Python 3.7, using the PyTorch 1.7.1 deep learning library and related development packages. The hardware environment for model training and testing was a regular GPU workstation with basic configurations such as an Intel Core i7-10700K CPU, 16GB of RAM manufactured by Samsung (Suwon-si, Republic of Korea), and a single Nvidia RTX 2080 Ti GPU (Santa Clara, CA, USA).

### 4.2. Comparative Experiment on Multi-Scale Feature Fusion and Extraction Module

**Figure 6:**
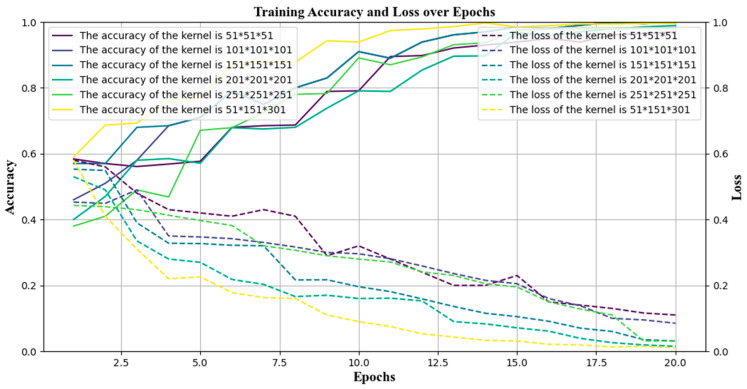
Comparative analysis of network training results for feature extraction and fusion with multiple convolution sizes.

To verify the effectiveness of the proposed fault diagnosis model, we separately validated the effectiveness of the multi-scale feature fusion module. Keeping other parts of the network structure unchanged, we compared the convolutional input of bearing data using various single convolution sizes—51, 101, 151, 201, 251, and 301—in the feature extraction one-dimensional convolution part. Each convolution size was trained for 20 epochs, and the training accuracy and loss graphs are shown in Figure 6.

As illustrated by the training results in Figure 6, the accuracy of feature extraction using a single convolution scale within the feature extraction and fusion network was generally suboptimal. The best single convolution scales were 151 and 201, achieving an accuracy of 99%, while the others fell below 98%. Our method was to employ three-channel parallelism, with each channel utilizing a distinct convolution kernel scale for feature extraction. The convolution kernel scales used were 51, 151, and 301, respectively, resulting in a model accuracy of 99.96%, the highest among all comparative experiments. It can be seen that the multi-scale feature extraction method with multi-channel fusion has obvious advantages. However, the size of the convolution kernel used for feature fusion and extraction is only a part of the network. The training of the network model also involves other hyperparameters, which have a certain coupling relationship. Determining the settings of these hyperparameters and their impact on network performance is a combinatorial optimization problem. Based on the Whale Optimization Algorithm (WOA) [32], we propose performing hyperparameter combinatorial optimization in the multi-scale feature extraction network to find the optimal solution in the training parameter space. The Whale Optimization Algorithm has the advantages of a fast convergence speed, strong global search capability, and simple and easy implementation. It is also relatively easy to use it for network hyperparameter optimization.

The network has three channels of one-dimensional convolution, with each channel’s convolution scale ranging from L ∊ [31, 501], the number of iteration cycles ranging from epoch ∊ [5, 50], the learning rate ranging from L_r ∊ [0.001, 0.3], the batch size ranging from batch ∊ [4, 128], and the number of heads in the Encoder’s multi-head attention mechanism ranging from M_head ∊ [6, 20]. Before optimization, we discretized the hyperparameter space into a series of fixed values, set the population size of the Whale Optimization Algorithm (WOA) to 30 and the maximum number of iterations to 100, and selected the fitness function as the mean squared error (MSE) to evaluate the function, thereby finding the optimal solution in the set of network hyperparameters. The hyperparameter values after optimization by the optimization algorithm are shown in Table 1.

Using the optimized hyperparameters to train the network resulted in fast convergence, high accuracy, and stability, with the model’s accuracy reaching 99.96%. Meanwhile, if the multi-scale feature extraction part of the network remained unchanged in the experiment and the multi-head attention encoding layer was pruned, the corresponding accuracy was only 68%. The experimental data demonstrate the effectiveness of the combination of the multi-head attention encoding layer and the multi-scale feature fusion network in fault diagnosis of bearing data. It can enhance the performance of the self-attention layer by assigning different representation subspaces to the attention layer and using multi-head attention to aggregate structural information before and after the data from the input, achieving a qualitative improvement in feature extraction performance.

### 4.3. Analysis of Transfer Task Results Using Different Methods

Data collected from simulated special vehicle rolling bearing test benches were used, with samples extracted from three different working loads denoted as domains A, B, and C. Each domain consists of three categories, comprising three types of defects and one normal condition, with 500 samples allocated to each category for the experiments and analysis of transfer tasks.

To evaluate the feature learning and generalization capabilities of different network architectures when applying transfer learning techniques, we selected the benchmark mainstream methods CNN [7], TCA [33], DDC [34], DAN [35], JAN [36], and DACNN [37] and compared them with our designed DNMFF. To ensure fairness in the comparison, all methods using convolutional neural networks to extract high-dimensional features were set to have the same network structure and parameters as the proposed method. Additionally, since unsupervised transfer component analysis (TCA) [33] is a shallow network structure with certain limitations in learning effective features directly from raw data, we needed to perform feature extraction on the raw data before applying this algorithm. This approach ensured that we fully leveraged the time–frequency characteristics of the data and enhanced the performance of the TCA algorithm in handling complex tasks. For deep domain confusion (DDC) [34], we followed the suggestions in the literature by using the maximum mean difference (MMD) to reduce the distance between fully connected layers in the CNN classification model. In the parameter settings for deep adaptation networks (DANs) and joint adaptation networks (JANs), we strictly followed the recommendations in references [36,38], adopting the multi-kernel maximum mean difference (MK-MMD) to minimize the distribution differences in the fully connected layers of the classification model. For the DAN, we used the joint maximum mean discrepancy (JMMD) to achieve the same objective. The application of these methods helped improve the model’s generalization ability across different data distributions, thereby enhancing classification performance. The domain adaptation convolutional neural network (DACNN) [37] employs adversarial training techniques in the source and target domains to achieve domain adaptation. This adversarial training method enables the DACNN to extract common features between the source and target domain data by confusing them and to learn a relatively optimal weight factor reflecting the ratio between the marginal and conditional distributions between the source and target domains, achieving good generalization performance across different domains.

To obtain reliable results, we conducted ten repeated experiments for each method and calculated the average accuracy as the final performance metric. This setup and experimental process helped us more accurately assess the performance of different methods, allowing us to draw more reliable conclusions. During the training process, we employed the Adam (Adaptive Moment Estimation) optimizer, which is an adaptive learning rate optimizer based on the gradient descent algorithm, with the number of iterations uniformly set to 200. To obtain reliable results, we performed 10 repeated experiments and calculated the average accuracy. The comparison results of the transfer tasks are shown in Table 2.

Based on the diagnostic results presented in Table 2, we can conclude the following: Compared to other domain adaptation diagnostic methods, the CNN demonstrates deficiencies in classification accuracy, achieving an average diagnostic accuracy of only 69.83%. Although CNNs can learn abstract high-dimensional feature representations, these features lack strong domain adaptation capabilities, resulting in poor accuracy when generalized to other tasks.

To improve classification accuracy, the TCA method adopts an approach that extracts 18 time domain and frequency domain features and combines them with the k-nearest neighbor (KNN) classifier method, achieving a higher average accuracy of 74.71%. This indicates that the TCA method has certain advantages in feature extraction and classification, but there is a significant difference in accuracy compared to domain adaptation diagnostic methods such as DDC, DAN, and DACNN. A joint adaptation network (JAN) is a network with domain adaptation capabilities that achieves unsupervised domain adaptation by matching the joint distributions of activations in multiple domain-specific layers. From the table, it can be seen that its performance is balanced and superior to other methods in transfer tasks. The advantage of this approach lies in not only adjusting the marginal distribution but also handling changes in the joint distribution through unsupervised domain adaptation. This method can be applied across different domains. In comparison, the DNMFF transfer diagnosis method based on multi-scale feature fusion has advantages in eliminating distribution differences between source and target domain data. It is a deep-learning-based cross-domain adaptive learning method with an average accuracy of 98.65% in six transfer tasks, capable of extracting superior feature representations and improving diagnostic performance. On the other hand, DNMFF adopts an independent encoding network structure that can adaptively align the marginal and conditional distributions between the source and target domains. It uses a weight factor to adaptively assign proportional relationships to these two distributions, thereby reducing the differences between source and target domain features and optimizing the network’s performance in transfer tasks.

### 4.4. Comparative Analysis of Feature Visualizations from Different Network Architectures

**Figure 7:**
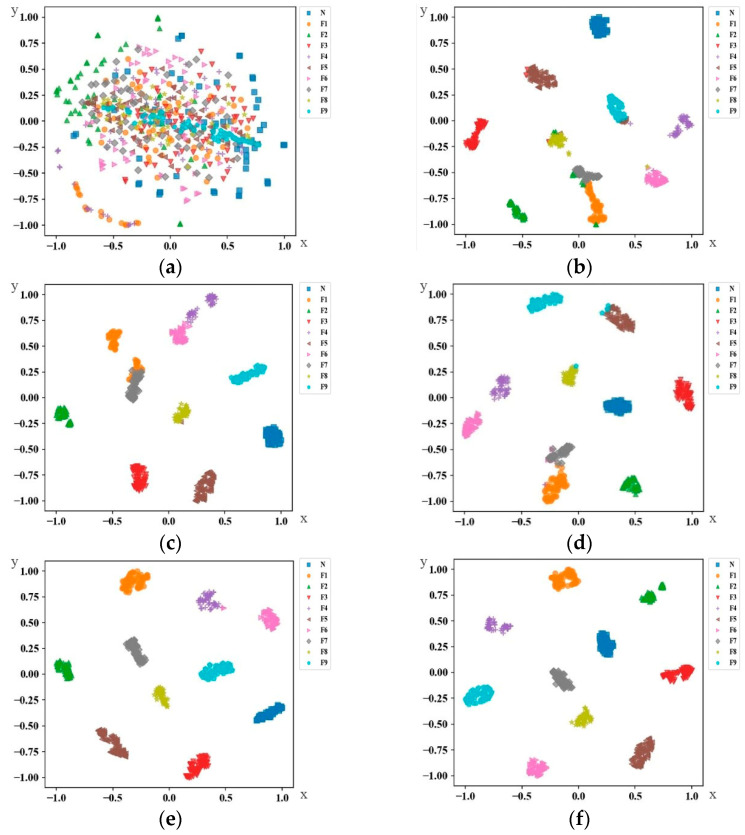
Visualization of the feature extraction results for the specialized vehicle bearing dataset in the migration task from C to B: (**a**) visualization of the feature extraction results from the input data; (**b**) employing a DNMFF with a single convolution scale of length 151; (**c**) substituting the encoding network in the multi-scale feature extraction network with an LSTM; (**d**) DAN; (**e**) DACNN; (**f**) DNMFF.

To demonstrate the effectiveness of each part of the DNMFF structure and the advantages of its overall performance, we conducted ablation comparison and horizontal comparison experiments with similar networks. Using domain B and C data from the simulation testbed as network inputs, we validated the network through the transfer task C→B. We applied the t-Distributed Random Neighbor Embedding (t-SNE) data visualization technique [39] to the fully connected layer before the network output layer, mapping the high-dimensional features of the entire connected layer onto a two-dimensional graph for visualization. The experiment setup and visualization content consisted of six parts, with the visualization results shown in Figure 7.

As shown in Figure 7a, the features of the original vibration signals overlap and are distributed in a chaotic manner in the two-dimensional space, with no clear boundary. Figure 7b,c show the results of ablation studies on the main components of the DNMFF. Figure 7b replaces the three-channel multi-scale convolution in the DNMFF with single-scale one-dimensional convolution operations. From the visualization results, it can be seen that points of the same category cluster together, but there is still a small area of overlap among the six fault categories between different samples. The dimensionality reduction results in the figure suggest that the overall accuracy after passing through the classifier would also be relatively low. Figure 7c shows the use of LSTM from reference paper [40] as a replacement for the encoding network in the multi-scale feature extraction module of DNMFF. The classification module consists of stacked LSTM neural networks for feature fusion and classification of one-dimensional temporal information. From the results shown in the figure, it can be seen that the features of each category are clustered closely together, indicating a relatively compact and impressive clustering effect. However, there is some overlap among the clusters formed by three fault categories, making the overall effect similar to that in Figure 7b. Figure 7d,e represent the DANs and DACNNs, respectively, which performed well in the transfer learning experiments. Both networks exhibit relatively good performance in the feature maps, with each category forming distinct clusters that are relatively far apart. Figure 7e shows that the domain-adaptive convolutional neural network can demonstrate the improvements brought by adaptation to existing transfer tasks and can better accommodate the differences in the current data domains. Compared to the DAN, it shows a certain degree of improvement, with larger inter-cluster distances. The results shown in Figure 7f are the best among the existing comparisons. Various categories form tight clusters that are relatively separated, with no overlap between clusters. The classification accuracy in the current data transfer is the highest, with relatively large distances between categories, indicating that training in transfer tasks is easier, and the classification performance within the same domain is very robust. Further analysis, based on comparing Figure 7b,f, revealed that the combination of multi-scale convolution and an attention mechanism can effectively allocate weights to features, enabling the extraction of high-level features and the extraction and measurement of domain features in the domain discriminator. The greater distance between the distributions of each category in Figure 7f indicates that the final classification layer is easier to train, resulting in optimal classification performance.

## 5. Conclusions

To improve the fault diagnosis capability for rolling bearings in special vehicles under cross-domain and cross-operating conditions, this study proposes the domain adversarial migration network for fault features (DNMFF), for rolling bearing fault identification. Unlike traditional domain adaptation diagnosis methods, the proposed network includes an independent multi-scale feature extraction and fusion network that learns invariant high-level feature representations from both source and target domain data. It can independently learn the features of each domain, reducing the differences in data feature distributions between the source and target domains, enhancing domain adaptation and diagnostic capabilities, and addressing the lack of generalization in traditional diagnostic models. To verify the effectiveness of the proposed algorithm, experiments were conducted using two bearing datasets. Firstly, we conducted multi-scale feature extraction experiments based on raw data, and according to the experimental results, the multi-scale feature fusion extraction method demonstrated significant advantages compared to single-scale feature convolution. Secondly, for the classification problem of unlabeled target domain data, we adopted a domain adversarial learning strategy to achieve the extraction of domain-invariant features between the source and target domains. Comparative experiments with traditional CNN and four domain adaptation diagnosis methods further verified the effectiveness of the proposed method. The method proposed in this paper achieves an average accuracy of 98.65% across six transfer learning diagnostic tasks, which was the highest among all the compared methods. In summary, this method improves the accuracy of cross-domain fault identification for rolling bearings and provides a general solution for bearing fault diagnosis in special vehicles under complex working conditions.

## Figures and Tables

**Table 1:** Optimization results for hyperparameters in multi-scale feature extraction network.

L	$L_{r}$	Epoch	Batch	$M_{head}$
51, 151, 301	0.01	20	16	16

**Table 2:** Comparison of results of transfer learning tasks.

Method	A→B	B→A	B→C	C→A	C→B	A→C	Average Value
CNN	66.61%	71.31%	67.33%	70.02%	71.16%	72.56%	69.83%
TCA	75.65%	78.55%	77.36%	71.25%	73.62%	71.83%	74.71%
DDC	91.78%	93.16%	94.68%	93.72%	95.16%	94.85%	93.89%
DAN	93.13%	95.57%	94.66%	95.61%	96.13%	93.76%	94.81%
JAN	93.36%	95.86%	93.76%	95.79%	93.56%	94.89%	94.54%
DACNN	90.56%	91.87%	92.16%	92.77%	92.35%	92.86%	92.10%
DNMFF	98.77%	99.15%	98.85%	98.78%	98.19%	98.17%	98.65%

## Data Availability

The original contributions presented in the study are included in the article, further inquiries can be directed to the corresponding author.
